# Functional analysis of *Salmonella* Typhi adaptation to survival in water

**DOI:** 10.1111/1462-2920.14458

**Published:** 2018-11-18

**Authors:** Robert A. Kingsley, Gemma Langridge, Sarah E. Smith, Carine Makendi, Maria Fookes, Tom M. Wileman, Moataz Abd El Ghany, A. Keith Turner, Zoe A. Dyson, Sushmita Sridhar, Derek Pickard, Sally Kay, Nicholas Feasey, Vanessa Wong, Lars Barquist, Gordon Dougan

**Affiliations:** ^1^ Quadram Institute Bioscience Norwich Research Park Norwich UK; ^2^ The Wellcome Trust Sanger Institute Wellcome Trust Genome Campus Hinxton, Cambridge UK; ^3^ The Westmead Institute for Medical Research, The University of Sydney, Sydney, Australia and Marie Bashir Institute for Infectious Diseases and Biosecurity The University of Sydney Sydney Australia; ^4^ Department of Medicine, University of Cambridge Addenbrooke's Hospital Hills Road, Cambridge UK; ^5^ Liverpool School of Tropical Medicine Liverpool UK; ^6^ Helmholtz Institute for RNA‐based Infection Research Würzburg Germany; ^7^ Faculty of Medicine University of Würzburg Würzburg Germany

## Abstract

Contaminated water is a major risk factor associated with the transmission of *Salmonella enterica* serovar Typhi (*S.* Typhi), the aetiological agent of human typhoid. However, little is known about how this pathogen adapts to living in the aqueous environment. We used transcriptome analysis (RNA‐seq) and transposon mutagenesis (TraDIS) to characterize these adaptive changes and identify multiple genes that contribute to survival. Over half of the genes in the *S.* Typhi genome altered expression level within the first 24 h following transfer from broth culture to water, although relatively few did so in the first 30 min. Genes linked to central metabolism, stress associated with arrested proton motive force and respiratory chain factors changed expression levels. Additionally, motility and chemotaxis genes increased expression, consistent with a scavenging lifestyle. The viaB‐associated gene *tviC* encoding a glcNAc epimerase that is required for Vi polysaccharide biosynthesis was, along with several other genes, shown to contribute to survival in water. Thus, we define regulatory adaptation operating in *S.* Typhi that facilitates survival in water.

## Introduction

Water is an important vehicle for the transmission of microbes. The enteric bacterial species *Salmonella enterica* is normally transmitted by the faecal–oral route from reservoirs of infected humans or animals through food and water. Water is of particular importance to the transmission of *S. enterica* serovar Typhi (*S.* Typhi), the aetiological agent of typhoid fever. Typhoid fever remains a significant cause of mortality and morbidity in low income countries, principally in Africa and Southeast and South Asia, with an estimated 20.6 million annual cases resulting in approximately 200,000 deaths (Buckle *et al*., 2012; Antillon *et al*., 2017; Kassebaum *et al*., 2017; Kim *et al*., 2017; Marks *et al*., 2017). The emergence and worldwide spread of multidrug resistant (MDR) *S.* Typhi, in part driven by the global spread of the H58 haplotype, has created enormous challenges for typhoid therapy and disease control in general (Wong *et al*., 2015; Klemm *et al*., 2018). Three vaccines are currently licensed for use in humans, two subunit vaccines composed of the Vi polysaccharide (ViPS) alone or conjugated to a carrier protein (YCV), and a live oral vaccine (Ty21a) (Mohan *et al*., 2015; Jeon *et al*., 2018). There remains interest in developing improved live oral vaccines strains such as M01ZH09 that is attenuated by mutations in *aroC* and *ssaV* genes (Darton *et al*., 2016).

Unlike most serovars of *S. enterica* where human infections are associated with zoonotic sources*, S.* Typhi is host‐restricted and causes disease exclusively in the human population and lacks a proven zoonotic reservoir (House *et al*., 2001). Environmental risk factors for typhoid include living in the vicinity of open wells or sewers, residence at low elevation and climatic events such as the rainy seasons (Sur *et al*., 2007; Wang *et al*., 2012; Corner *et al*., 2013; Dewan *et al*., 2013; Akullian *et al*., 2015). The greatest risk of transmission is in densely populated areas and settings that lack sanitation and access to clean drinking water (Vollaard *et al*., 2004; Hosoglu *et al*., 2006). Indeed, transmission through consumption of contaminated drinking water is a greater risk factor than through direct human to human contact (Baker *et al*., 2011). Environmental sources especially water contaminated with human faeces are, therefore, important factors driving persistence and transmission.

*S.* Typhi is notoriously difficult to culture directly from water sources where contamination is suspected on epidemiological grounds (Cho and Kim, 1999; Karkey *et al*., 2016). The length of time *S.* Typhi is able to survive in a water environment or which genes contribute to survival is also not well understood. However, for consumption of contaminated water to be a risk factor for typhoid, *S.* Typhi must retain viability and potential to transmit to the next human host following excretion into water. We therefore address the hypothesis that entry into water is accompanied by an adaptive transcriptional response involving genes that contribute to survival in water or revival from the water environment.

## Results

### S. *Typhi entry into water results in adaptive transcriptional changes in central metabolism and response to stress*

**Figure 1:**
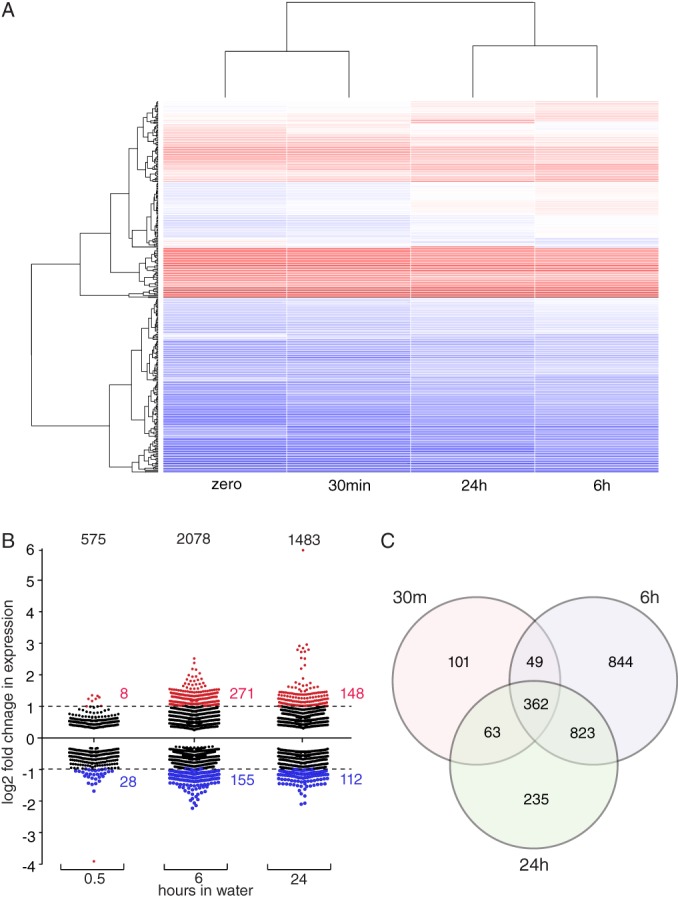
Summary of transcriptional response of *S.* Typhi on entry into water following culture in rich medium. A. Unsupervised hierarchical clustering of normalized (baseMean) sequence reads mapped to 239 genes that were significantly differentially expressed (*p* < 0.05) > two‐fold, for at least one time point after entry into the water environment compared with culture in broth. Heat map indicates highly (red) and lowly (blue) expressed genes.B. Each point indicates the fold change difference in expression of a gene compared with expression during culture in mid log phase in broth.C. Venn diagram indicating the number of genes with significantly altered expression.

We analyzed the transcriptional response of *S.* Typhi BRD948 after transferring this isolate from broth culture into water. Pure water was used as a well‐defined environment to ensure reproducibility. To this end, total RNA was prepared from *S.* Typhi grown to late exponential phase in LB broth, and after 0.5, 6 and 24 h (h) incubation in water. A total of 2477 genes (57% of the analyzed features) exhibited significantly altered relative expression (*p* < 0.05) at one or more of the time points in water compared with culture in LB broth, with just 1841 genes exhibiting no significantly change at any time point (Supporting Information Table S1). The mean fold‐change in expression was 1.46, 1.55 and 1.57 for genes with significant decreased expression and 1.36, 1.67 and 1.57 for genes with increased expression at 0.5, 6 and 24 h respectively. Unsupervised hierarchical clustering of the expression data indicated that profiles during culture in LB broth and after 0.5 h in water were more closely related to one another than profiles at 6 and 24 h (Fig. 1A). Relatively few genes exhibited altered expression at 0.5 h, with a greater response by 6 and 24 h after entering the water environment (Fig. 1B and C). A total of 575 genes exhibited a significant change in expression after 0.5 h and this increased to 2082 after 6 h then decreased to 1483 at 24 h. There was considerable overlap in genes with altered expression at both 6 and 24 h, with 362 genes exhibiting altered expression at all time points in water compared with LB broth. Consistent with the absence of a functional *rpoS* gene in S. Typhi Ty2 due to a frameshift mutation (Burda *et al*., 2018), expression of genes in the RpoS regulon (Patten *et al*., 2004) were generally not affected by entry into water.

One of the greatest impacts of exit from LB broth (and indeed the intestine) into water is a dramatic change in nutrient availability. We therefore superimposed significantly differentially expressed genes onto a metabolic model of *S.* Typhi composed of 4353 polypeptides in 354 pathways in the biocyc database (Caspi *et al*., 2016) (Supporting Information Fig. S1). After 0.5 h in water, genes were significantly over represented (*p* < 0.01) in pathways involved in central metabolism (glycolysis and the TCA cycle) that produce precursor metabolites for cellular biosynthesis. Genes involved in the TCA cycle (*acnA, acnB, sucA, icdA, sucB, sucC, sucD, fumA* and *fumC*), components of pyruvate dehydrogenase that links glycolysis with the TCA cycle (*aceE* and *aceF*), and enzymes participating in the respiratory electron transfer chain (*icdA, cyoB, cyoC, cyoD, cydA, phsA, phsC, glpC*) all exhibited increased expression. The *eutE* gene exhibited the greatest increase in expression after 0.5 h, with a five‐fold increase at this time point. EutE is an aldehyde dehydrogenase that converts acetaldehyde to acetyl‐CoA destined for the TCA cycle. The *eutE* gene remained highly up‐regulated at the 6 and 24 h time points, suggesting that acetaldehyde, which is a widely distributed aldehyde in nature (Dabrowska *et al*., 2014), may be an important potential source of energy for *S.* Typhi in the environment.

Thus, genes involved in central metabolism that were differentially expressed, were generally upregulated in the first 0.5 h. One of the few genes involved in central metabolism that was significantly down‐regulated at this time point was *tktB*, which decreased nearly two‐fold. TktB is thought to be a transketolase (Sprenger *et al*., 1995), which are key enzymes required for catabolism of pentose sugars, and links the pentose phosphate pathway to glycolysis (Vimala and Harinarayanan, 2016). Conversely, expression of a second putative transketolase encoded by *tktA* was relatively increased at all time points in water compared with LB broth (Supporting Information Table S1). This is consistent with reports that in *Escherichia coli* these isoenzymes are differentially expressed in response to osmotic stress (Sprenger *et al*., 1995). Most of the genes involved in central metabolism initially upregulated after entry into water continued to be expressed at similar levels at 6 and 24 h.

At 6 and 24 h, some genes involved in the degradation of substrates that serve as sources of nutrients and energy utilization, exogenous sources of essential metabolites or assimilation of essential compounds had altered expression levels. For example these included genes involved in carbohydrate and carboxylate degradation pathways (*xylA*, *xylB*, *malS*, *malQ*, *fucI*, *fucK*, *rhaA* and *glgP*). In addition to nutrient stress, other stress characteristics include osmotic stress and the maintenance of the proton motive force. In this regard, the *pspA*, *pspB* and *pspC* genes were among the most highly upregulated genes at 24 h after entry into water. These genes encode phage shock proteins implicated in survival under stress conditions and that function in the maintaining proton motive force (Kleerebezem *et al*., 1996; Dworkin *et al*., 2000). The *osmB* gene, which encodes a lipoprotein induced by osmotic stress, was also highly upregulated at this time point (Jung *et al*., 1990; Charoenwong *et al*., 2011).

### *Entry into water results in changes to expression of motility and virulence genes*

**Figure 2:**
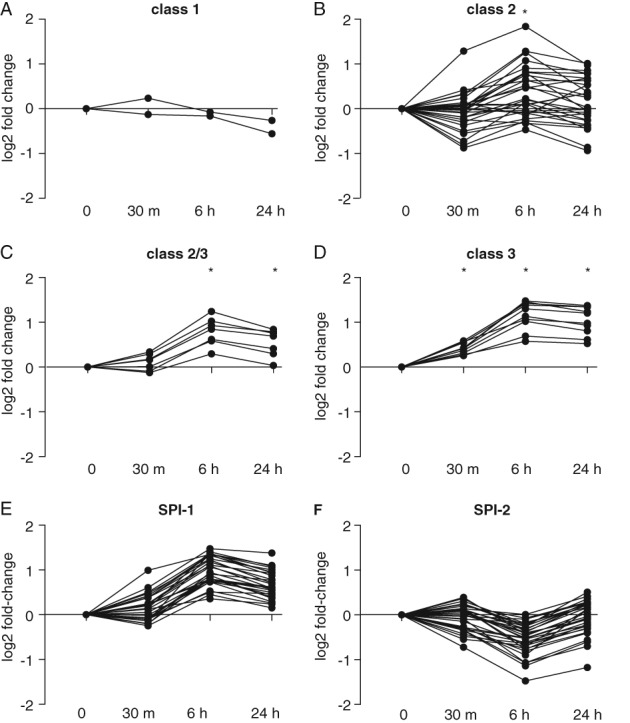
Transcriptional response of motility and pathogenicity island genes on entry into water. Fold‐change (log2) in expression of flagella and chemotaxis genes at 0.5 h (30 min), 6 h (6 h) and 24 h (24 h) after entry into water, are indicated at each time point relative to expression during culture in LB broth (0 on the y‐axis) for, class 1 (A), class 2 (B), class 2 and 3 (C) and class 3 (D) promoters, and for SPI‐1 (E) and SPI‐2 (F) encoded genes.

*S.* Typhi encode flagella biosynthesis, motility and chemotaxis genes in multiple operons and clusters, whose transcription is split into a hierarchy based on three promoter classes (Chevance and Hughes, 2008). We observed class‐specific transcriptional responses that were consistent with increased motility on entry into water (Fig. 2A–D). Entry of *S.* Typhi BRD948 into water was characterized by a strongly coordinated increased expression of genes from class III flagella promoters, while genes from class I exhibited little change. The expression of class II flagella genes appeared relatively discordant with some exhibiting moderate up and others moderate down regulation. These data are consistent with the scenario in which the hook‐basal body substructure was largely preformed and entry into water resulted in increased expression of components required for a functional flagellum with motor activity, combined with a full repertoire of chemotaxis function. This would permit movement to sources of nutrients.

*Salmonella* pathogenesis involves two type III secretion systems (TTSS) encoded on *Salmonella* pathogenicity islands (SPI) 1 and 2. After 0.5 h in water, there was relatively little change in expression of genes on either island, but after 6 h SPI‐1 genes exhibited a mean increase in expression in excess of two‐fold while SPI‐2 genes were expressed at a lower level. After 24 h SPI‐1 genes remained at a comparatively higher expression level, while SPI‐2 genes returned to a similar level of expression to that detected at 0.5 h (Fig. 2E and F).

### *A subset of* S. *Typhi genes are essential for optimal survival in water*

In order to identify *S.* Typhi genes that are involved in survival in and revival from water we performed a transposon directed insertion site sequencing (TraDIS) screen using a Transposon 5 (Tn5) insertion library harbouring approximately 250,000 unique insertion sites. The composite mutant library harboured, on average, a Tn5 insertion every 20 bases in the genome (Langridge *et al*., 2009). The insertion library was harvested from LB broth, suspended in water at a density of approximately 5 × 10^7^ cfu ml^−1^ and incubated without shaking at 28°C for 24 h. The number of viable *S.* Typhi decreased within 24 h to 7.5 × 10^5^ cfu ml^−1^. The relative impact of insertions in each gene for survival and subsequent revival from water was determined by quantification of transposon insertion sites in the amplified library before exposure to water (input pool) and after re‐culturing from water (output pool).

**Figure 3:**
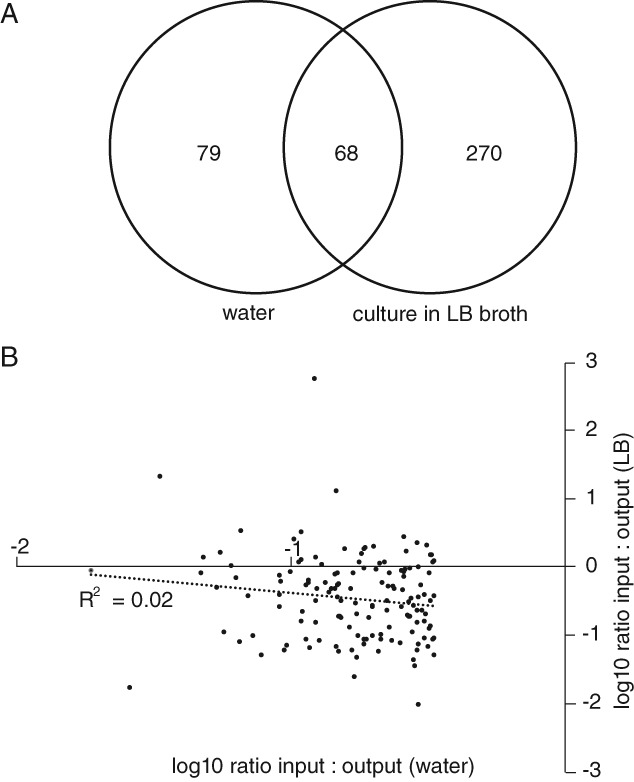
Comparison of candidate essential genes for optimal growth in LB broth and survival in water.A. Venn diagram indicating the union of genes with TraDIS reads greater than three‐fold mapped in the input compared with output pools following culture in LB broth or in water for 24 h.B. The ratio (log10) of mapped reads in the output pool compared with the input pool following culture in LB broth or in water for 24 h. The regression line and R square are indicated.

A total of 248 053 transposon insertion sites were identified in the input pool from 3 428 702 mapped sequence reads. The output pool contained 172 399 insertion sites identified from 3 841 843 mapped sequence reads. In order to determine the relative density of Tn5 insertions in each gene in the input and output libraries we determined the number of uniquely mapped reads per gene after normalization (Supporting Information Table S2). A total of 147 genes had greater than three‐fold more reads per gene in the input relative to the output pool and were considered candidate genes, essential for optimal survival and or revival from water. There was little correlation between genes for which insertions were counter‐selected in water and those previously reported for LB broth culture (Barquist *et al*., 2013), indicating distinct modes of survival and replication in these two environments (Fig. 3). Of the 147 candidate genes implicated in water survival and revival, 68 also exhibited an influence on culture in LB broth, while 270 genes essential for growth in LB broth were not required for survival in water.

**Figure 4:**
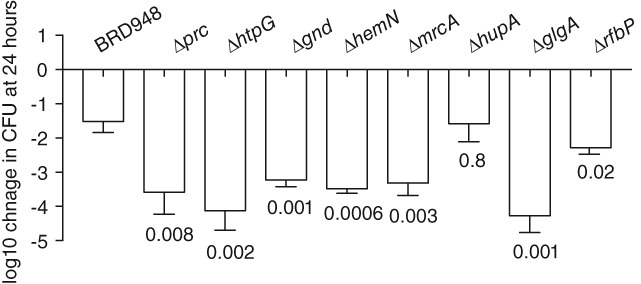
Survival of *S.* Typhi BRD948 and derived mutants in water following incubation for 24 h. The mean log10 fold‐change in cfu at 24 h relative to time zero are plotted (open bar) with the standard deviation of six biological replicates. The *p*‐value (Student's *t*‐test) are indicated for comparison of each mutant derivative with the parent BRD948.

In order to determine whether genes identified by the TraDIS screen influenced viability in water, we constructed null mutant derivatives in nine genes and tested these for viability in water (Fig. 4). Here, the *prc*, *htpG*, *gnd*, *hemN*, *mrcB*, *hupA*, *glgA*, *tviC* or *rfbP* were replaced in *S.* Typhi BRD948 with a DNA cassette encoding the *cat* gene, conferring chloramphenicol resistance. In the TraDIS experiment these genes had mapped reads ranging from a log ratio of input: output of approximately 5 (*hemN*) to 50 (*rfbP*). A decreased survival in water was observed for all these *S.* Typhi BRD948 mutant derivatives except one, *hupA.* While 24 h in water resulted in a 1.5 log decrease in viable bacteria of the parent BRD948, all mutant derivatives except *hupA,* decreased by 3.2–4.3 log.

Ten mutants that were under‐represented after selection in the TraDIS screen encoded enzymes involved in central metabolism and the respiratory chain (Supporting Information Fig. S1). These included mutants in genes encoding enzymes in the TCA cycle and acid fermentation (*acnA* and *fumC*), acid fermentation alone (*ackA*), glycolysis (*pfkA* and *gpmA*), and the pentose phosphate pathway (*gnd, zwf* and *ybhE*). A number of mutants for components of the respiratory chain were also under‐represented, including *cyoC*, *cyoD* and *nuoL*, and genes required for biosynthesis of heme and heme‐like prosthetic groups (*hemN, hemY* and *hemX*), also involved in the respiratory chain (Choby and Skaar, 2016). Other pathways with clusters of multiple gene hits that were under‐represented were involved in glycogen metabolism, including glycogen biosynthesis genes (*glgA*, *glgB* and *glgC*), and glycogen degradation genes (*glgP* and *glgX*) (Supporting Information Fig. S1). Several genes encoding enzymes involved in fatty acid oxidation (*fadB*, *fadD*, *fadE*, *fadJ* and *fadL*) were also under‐represented. Many mutants that were under‐represented in the output pool were in genes involved in the envelope of the outer membrane, peptidoglycan and inner membrane. For example, genes involved in biosynthesis of the lipopolysaccharide (LPS), including *rfbP*, *rfc*, *waaI*, *rfbE*, *galE*, *rfbD*, *rfbU*, *waaL*, *rfbM*, *rfbF* and *waaP*, and *mrcA*, *mrcB* and *prc* genes involved in peptidoglycan biosynthesis. Two regulators were under‐represented, a two‐component system family regulator encoded by *sirA*/*barA*, and a key regulator of oxidative stress, directed by *oxyR* (Tartaglia *et al*., 1989). As might be expected for selection of functions outside of the host, insertions in virulence genes, and motility and chemotaxis were not under‐represented in the output pool after 24 h in water.

**Figure 5:**
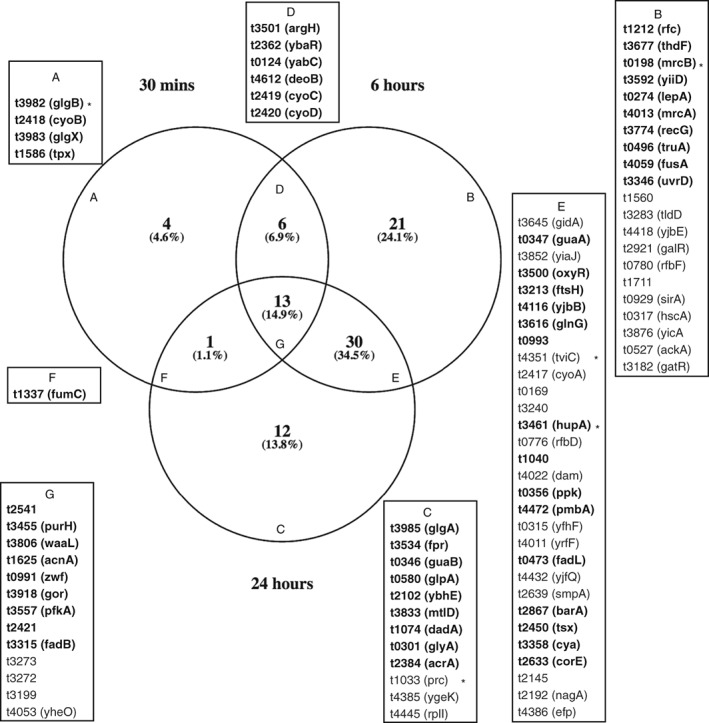
Comparison of candidate essential genes for survival and revival in water and differentially expressed genes on entry into water. The Venn diagram indicates the union of genes identified by TraDIS (reads IN = 20: reads OUT +20 with genes differentially expressed (false discovery rate < 0.05 and log_2_ fold‐change > 2) at 0.5, 6 and 24 h after entry into water. Genes in each sector are identified in boxes with genes that exhibited increased expression (bold text) or decreased expression (grey text) reported.

Of the 147 candidate‐genes essential for optimal survival and or revival from water, 87 exhibited altered expression at one or more time points (Fig. 5). In order to determine if genes with increased or decreased expression at each time point were more likely to be required for optimal survival in water as identified in the TraDIS screen, we tested for enrichment using a hypergeometric distribution test. Genes with increased expression at 0.5 or 24 h after entry into water were more likely to be within the subset of 176 putative essential gene. A similar enrichment was not observed for genes that were expressed at a lower level at these time points or genes differentially expressed at the 6 h time point. Of 279 genes that exhibited increased expression after 0.5 h in water, 20 were also under‐represented in the TraDIS experiment, significantly more than would be expected by chance (*p* = 0.0003). Similarly, of 766 genes that exhibited increased expression after 24 h in water, 34 were putative water survival genes, again greater than would be expected by chance (*p* = 0.032).

### *The TviC glcNAc epimerase but not Vi polysaccharide is required for survival in the water environment*

**Figure 6:**
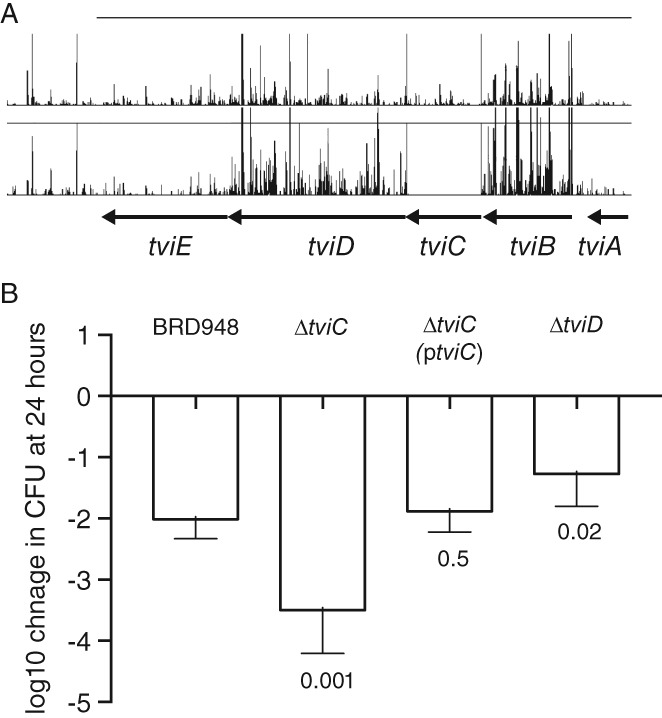
Evidence for differential role of *tviC* and *tviD* genes for survival in water.A. The number of TraDIS reads that map to insertion sites in the viaB locus. The height of the vertical lines is proportional to the number of site specific reads with the maximum representing 200 mapped reads. Genes are indicated as solid arrows.B. The mean log10 fold‐change in cfu at 24 h relative to time zero is plotted (open bar) with the standard deviation of six biological replicates. The *p*‐value (Student's *t*‐test) is indicated for comparison of each mutant strain with the parent BRD948 strain.

A striking observation in the TraDIS screen was the relative paucity of transposon insertions in the *tviC* gene in the output pool, but abundance of insertions in *tviA*, *tviB*, *tviD* and *tviE* genes (Fig. 6). In the input library 1014 reads were mapped to 134 insertion sites in the *tviC* gene, while just seven reads were mapped to two sites in the output pool (Supporting Information Table S2). In contrast, in the *tviD* gene, 7390 reads were mapped to 448 insertion sites in the input pool and 16 931 reads were mapped to 438 sites in the output pool. All of the *tvi*ABCDE genes are present in an operon where they are expressed from a common promoter and regulated by TviA. They encode enzymes involved in the biosynthesis of the Vi polysaccharide, a homopolymer composed of α‐1,4 (2‐deoxy)‐2‐*N*‐acetyl‐3‐*O*‐acetylgalacturonic acid. *tviC* encodes a UDP‐GlcNAcA epimerase essential for biosynthesis of the monomer UDP‐GalNAcA, from which the Vi is polymerized. Deletion of *tviC* resulted in a decrease in survival in water after 24 h by a factor of about 20. Replacement of a functional *tviC* gene with expression driven from the native promotor on a plasmid resulted in survival similar to that of *S.* Typhi BRD948. However, deletion of *tviD* resulted in an approximately 10‐fold increase in survival in water. Neither deletion of *tviC* nor *tviD* resulted in changes in utilization of 192 carbon sources determined using a phenotyping microarray. These survival data are consistent with the TraDIS screen data and suggested that expression of Vi is not required for survival in water, but expression of the TviC UDP‐GalNAcA epimerase significantly increases survival.

## Discussion

Despite many epidemiological studies reporting the importance of water in the transmission of *S.* Typhi (Vollaard *et al*., 2004; Hosoglu *et al*., 2006; Sur *et al*., 2007; Wang *et al*., 2012; Corner *et al*., 2013; Dewan *et al*., 2013; Akullian *et al*., 2015), comparatively little is known about the environmental survival strategies employed by this pathogen. In this study, we determined the transcriptional response of *S.* Typhi BRD948 to moving into water and identified multiple genes that contribute to survival. Our data reveal a dynamic transcriptional adaptation process that continued past 6 h, which involves an altered expression of 57% of annotated genes of *S.* Typhi. The peak of transcriptional change occurred between 0.5 and 6 h after entry into water. Despite the sudden shock of transfer from culture medium to water, the number of genes with altered expression after 0.5 h was modest, with 575 exhibiting a statistically significant change. In contrast at 6 h, a total of 2078 genes had changed expression. These relatively fluid changes in gene expression suggest that there are numerous adaptations that prepare the *S.* Typhi bacterium for survival in water.

A total of 362 genes exhibited altered relative expression at all time points monitored after entry into water. These core genes encoded enzymes in metabolism including biosynthesis and degradation of substrates of central metabolism. This is consistent with entry into a nutrient poor environment and likely reflects in part the need to channel precursor carbohydrate and carboxylate molecules into pathways for the generation of substrates for respiration in order to maintain the proton motive force. Genes involved in central metabolism were particularly prominent in the first 0.5 h, while at later time points there was a broader change in expression pattern. As a general trend, genes involved in biosynthesis were more likely to exhibit decreased expression and genes involved in degradation more likely to be increased in expression, particularly at the 6 and 24 h time points.

Transmission of bacteria via the environment depends on the survival of highly stressed organisms that are nonetheless resuscitated in vivo and lead to a productive infection (Colwell *et al*., 1996). The *S.* Typhi in this study exhibited signs of significant stress indicated by the increased expression of genes associated with maintainence of the proton motive force. Limited availability of iron, a co‐factor in multiple enzyme reactions (Kingsley *et al*., 1995), in water is likely to be a significant stress factor (Reissbrodt *et al*., 2000). Indeed, supplementation of culture media with ferrioxamine E, an exogenous siderophore able to provide iron to *Salmonella*, resulted in increased resuscitation from Soil and water microcosms (Reissbrodt *et al*., 2000). Consistent with this, we observed increased expression of genes involved in the biosynthesis and transport of the endogenous siderophore enterochelin (*entD*, *entF*, *fepB fepD*, *fepG* and *fepC*), after 6 h in water. Although mutations in these genes were not significantly under‐represented after selection in the TraDIS screen, this may be because intracellular reserves of iron were sufficient for resuscitation due to the relatively short time in the water used in our experiments.

With the exception of one gene involved in the pentose phosphate pathway, all genes that were differentially regulated and encoding enzymes in central metabolism were increased in expression at all time points. Consistent with the likely importance of central metabolism, a large number of candidate water survival genes were identified in the TraDIS screen, including examples encoding enzymes of the TCA cycle, glycolytic pathways, pentose phosphate pathway, fermentation and electron transfer in the respiratory chain.

The Vi polysaccharide is an important virulence determinant of *S.* Typhi, but little is known about any role in the external environment (Raffatellu *et al*., 2005; Janis *et al*., 2011; Jansen *et al*., 2011). Our data suggests that expression of Vi polysaccharide biosynthesis genes may compromise the ability of *S.* Typhi to survive in water, possibly as Vi expression imparts a metabolic burden. Consistent with this, the genes of the ViaB locus were down‐regulated on entry into water. Despite this, one of the genes, *tviC*, was actually required for optimal survival in water. Since all other Vi polysaccharide genes (on the viaB locus) are dispensable for survival in water, it is likely that the *tviC*‐encoded UDP‐GalNAcA epimerase activity plays an additional role. It could be that *S.* Typhi *tviC* mutants are less stable in water due to factors such as osmotic stress or that this enzyme is required for some other unknown biosynthetic pathway. However, to date we have been unable to identify this despite using extensive phenotyping approaches.

Survival in and revival from water is a critical characteristic of the transmission of *S.* Typhi and further studies are required to investigate the survival in polluted and brackish water more akin to the natural environment are required. An understanding of the molecular basis of this phase of the life cycle has potential applications in the control of transmission during natural infections, but also to improve the acceptability of live oral vaccines. Inadvertent vaccination during mass‐immunization campaigns has been documented for live oral vaccines, with potential health impacts of live oral *S.* Typhi vaccines on immunocompromised people. We have previously proposed inactivation of intestinal colonization and persistence factors as a potential route to decreasing the likelihood of transmission of live vaccine strains (Abd El Ghany *et al*., 2007). This study provides the knowledge base for the rational design of improved live oral vaccines with additional safe guards affecting environmental survival to further improve the acceptability of live oral vaccines.

## Experimental procedures

### *Bacteria and mutant construction*

*S.* Typhi BRD948 is a derivative of strain Ty2 (Tacket *et al*., 1997). The isolate was routinely cultured in Luria Bertani (LB) Broth supplemented with 0.004% phenylalanine, 0.004% tryptophan, 0.001% para‐aminobenzoic acid, 0.001% dihydrobenzoic acid and 0.004% tyrosine (aro‐mix). *S.* Typhi mutant derivatives harbouring targeted independent mutations in the genes *prc*, *htpG*, *gnd*, *hemN*, *mrcB*, *hupA*, *glgA*, *tviD*, *tviC* or *rfbP* were constructed by replacing the wild type gene with a DNA cassette encoding a chloramphenicol resistance gene (*cat*) by allelic exchange using recombineering based on a method described previously (Perkins *et al*., 2013). Deletion of the gene in each case was from the ATG start to the stop codon reported in the whole genome sequence annotation (Parkhill *et al*., 2001). For construction of non‐polar deletion derivatives, the *cat* gene was removed by introduction of plasmid pCP20 that encodes a site‐specific FLP recombinase as described previously (Datsenko and Wanner, 2000). For construction of a *S.* Typhi derivative in which the *tviC* deletion was complemented by a wild type copy of the *tviC* gene under the control of the native *viaB* locus promoter on a plasmid, the promoter and *tviC* gene were amplified by PCR and spliced using overlap extension PCR using the oligonucleotide primers: 5′‐ttcgtaagccgtcatgaagtctccttatgctgaaataac‐3′ and 5′‐ataAAGCTTaacatctagcgagaaaatattttg‐3′ and 5′‐ataGAATTCagttataccgaggaatacaaagtag, 5′‐gcataaggagacttcatgacggcttacgaagaactac‐3′, for the promoter and *tviC* gene respectively.

### *RNA preparation and sequencing*

*S.* Typhi BRD948 was cultured in LB supplemented with aro‐mix in 10 ml volume at 37°C with shaking to an OD_600nm_ of 2 (stationary phase). About 2 ml of culture was harvested for RNA preparation or washed once in RO water and suspended in RO water to a final concentration of 1 × 10^6^ cfu ml^−1^ and incubated at 28°C for 30 min, 6 h or 24 h. Three biological replicates with different initial cultures of bacteria were performed for each time point. For preparation of RNA, bacteria were harvested by centrifugation, the supernatant removed by aspiration and the pellet immediately frozen in liquid Nitrogen. The harvest process was complete within 5 min in order to minimize changes in gene expression. Total RNA was prepared using the FastRNA PRO Blue Kit (MP biomedicals), DNA removed by treatment with 2 units of TURBO DNA‐free DNAase (Ambion), reverse transcribed using Superscript III reverse transcriptase (Invitrogen) and sequenced using Illumina HiSeq 2000 sequencer with a read length of 100 nucleotides in 36‐plex on a single lane. Sequence reads were mapped to the *S.* Typhi Ty2 genome sequence (acc. No. AE014613) using Bowtie 2, and RPKM values determined using Artemis software (Carver *et al*., 2005). The R package DESeq, was used to implement negative binomial distribution statistics for RNA‐seq data. A false discovery rate of 0.05 was considered statistically significance for differentially expressed genes. Ribosomal genes and repeat sequence were filtered out from final tables. The relationship between expression profiles was initially investigated by unsupervised hierarchical clustering by generation of a matrix of Euclidean distances from normalized read counts, using the *heatmap* function of the R stats package (Bodenhofer *et al*., 2011). The RNA‐seq data has been deposited in the ArrayExpress database at EMBL‐EBI http://www.ebi.ac.uk/arrayexpress (accession no. E‐MTAB‐7160).

### *TraDIS library and survival in water*

The high‐density *Tn*5‐based transposon insertion library in a derivative of *S.* Typhi strain BRD948 has been described previously (Langridge *et al*., 2009). About 2.1 × 10^9^ cfu of this transposon library containing, on average, more than 5000 cfu for each insertion site, was used to inoculate 100 ml Luria Broth + aro‐mix and incubated at 37°C with shaking for 16 h. Bacteria from 40 ml of the subsequent culture was harvested by centrifugation, resuspended in 35 ml of sterile reverse osmosis (RO) purified water and 10 ml aliquots were used to inoculate 990 ml of sterile RO purified water. About 10 ml of the input library culture was also added to a fresh 100 ml and cultured at 37°C with shaking for 16 h. The cfu in each flask of the library in water were determined by serial dilution and culture on LB agar + aro‐mix. On day two, bacteria in 40 ml of water (~ 3 × 10^7^ cfu) was harvested by centrifugation and cultured in 100 ml LB broth at 37°C with shaking for 16 h. In this way both the input and output libraries were cultured in LB + aro‐mix and therefore changes in the relative frequency of insertions in each gene was due to survival in water and subsequent revival from water. Genomic DNA were purified from 1 ml of culture using a Wizard kit (Promega) according to manufacturer's instructions. Genomic DNA was fragmented, transposon insertion sites amplified and libraries prepared for Illumina HiSeq 2500 with modifications and sequence adjacent to *Tn*5 insertion sites determined as previously described (Langridge *et al*., 2009). Ratios between input and output read counts per gene were calculated as a proxy for changes in mutant abundance. A pseudocount of 20 was added to both the numerator and denominator of the ratio to avoid instability in low count genes and provide a conservative estimate of the change in mutant abundance.

### S. *Typhi metabolic pathway database construction and analysis*

Using Pathway Tools software (Karp *et al*., 2002), a pathway/genome (PGDB) was generated using the genome sequence and annotation of *S.* Typhi CT18 (accession AL513382). The PGDB was manually curated in accordance with software instructions including deletion of falsely predicted pathways and hole‐filling (Green and Karp, 2004). The *S.* Typhi PGDB from Pathway Tools version 20.5 contained 1055 enzymes and 186 pathways. Differentially expressed genes (RNA‐seq) and essential genes (ratio < 0.3 in TraDIS analysis) in metabolic pathways were analyzed for over‐representation in pathways was tested using the Fisher Exact test with Bonferroni correction for multiple tests.

### *Metabolic profile by phenotyping microarray*

Respiration of *S.* Typhi BRD948, BRD948 Δ*tviC::cat* mutant and BRD948 Δ*tviD::cat* mutant strains in the presence of 192 carbon sources was determined using microarray plates PM1 and PM2 (Biolog). *S.* Typhi strains were grown overnight at 37°C from frozen stocks on LB containing aro‐mix. Colonies were streaked onto fresh LB‐Aro plates before inoculation into 15 ml IF‐0a minimal medium (Biolog) to 42% transmittance measured by a turbidometer (Biolog). About 20 ml of IF‐0a + dye B (1.6% v/v) were prepared with Aro‐mix added at 1:50 of normal concentration. About 4 ml of inoculum was added to the 20 ml dye solution, and 100 μl of the solution was added to each well of a PM‐1 and PM‐2 plate, one each per strain.

## Supporting information


**Fig. S1.** Metabolic pathways map showing changes in genes expression at 0.5, 6 and 24 h after entry into water. Genes encoding enzymes with significant change in expression ranging from ≤2‐fold (green) to ≥2‐fold (red) on entry into water.

**Table S1.** RNA‐seq processed data.

**Table S2.** Processed TraDIS data.
