# Supplementary figures and images for: Functional analysis of Salmonella Typhi adaptation to survival in water
Source: Environ Microbiol. 2018 Nov 18;20(11):4079–90. doi: 10.1111/1462-2920.14458 (PMC6282856; doi:10.1111/1462-2920.14458)

Metabolic pathway overview

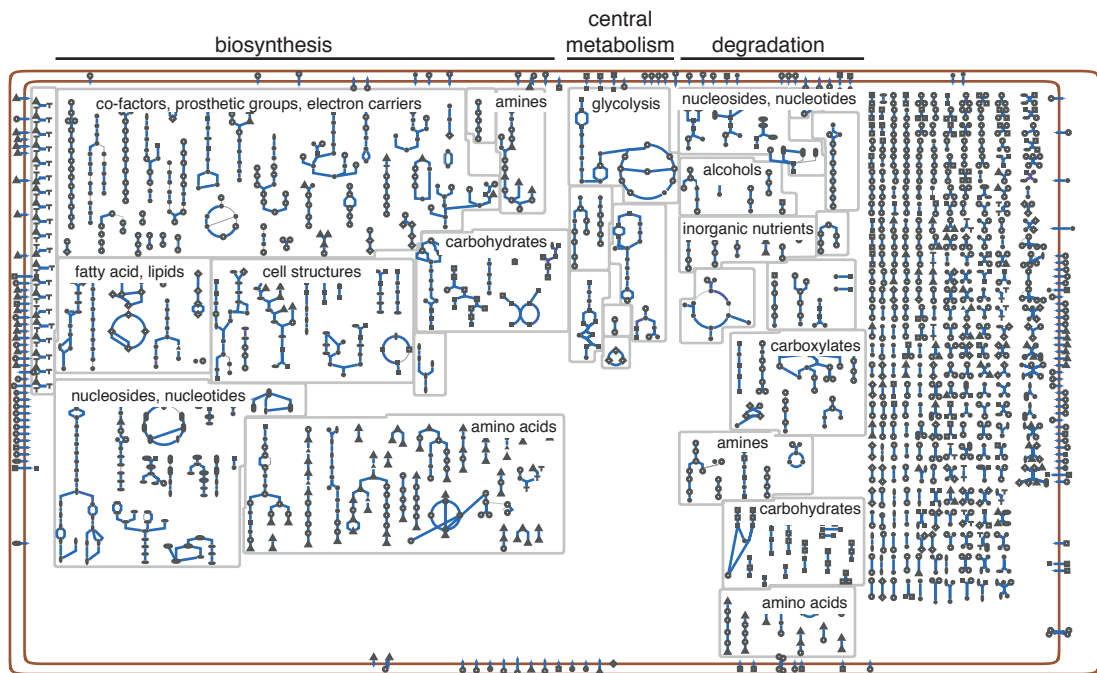

30 minutes

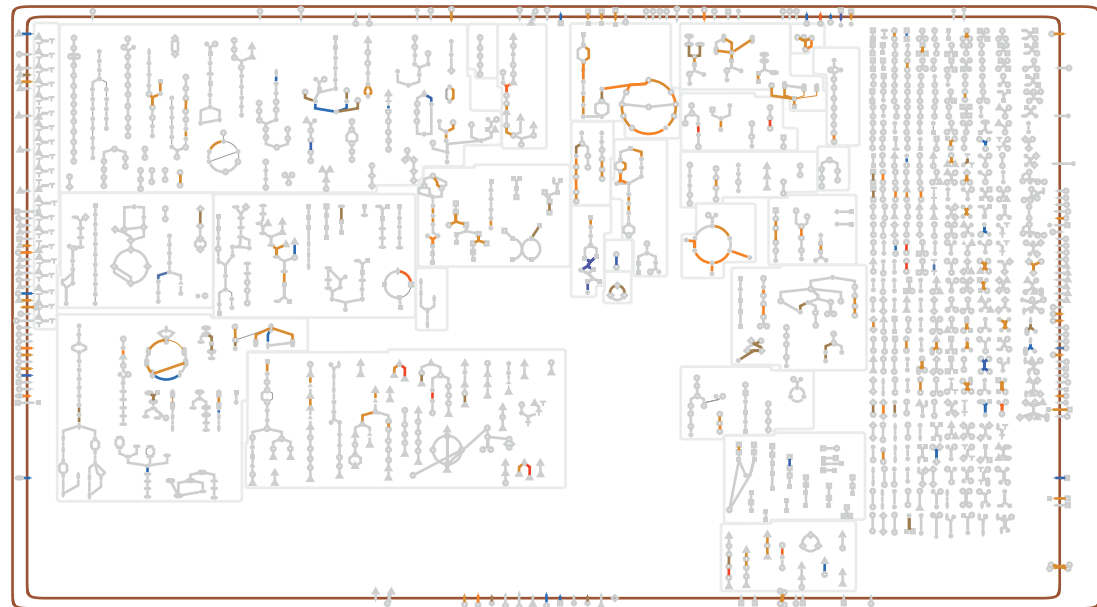

0.5h

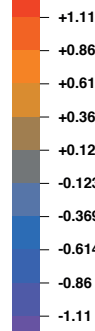

6 hours

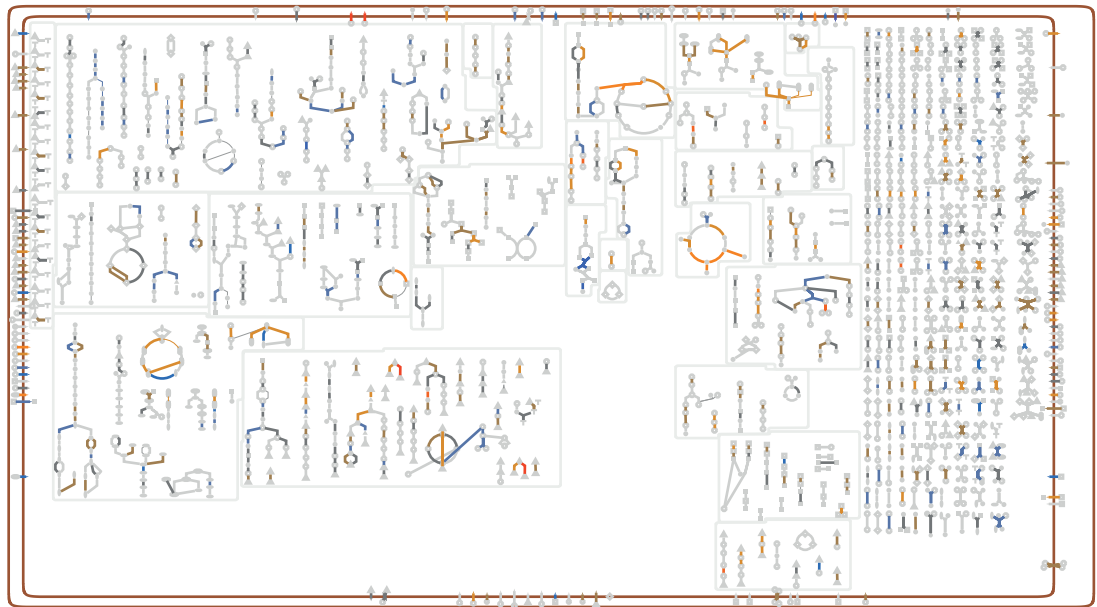

6h

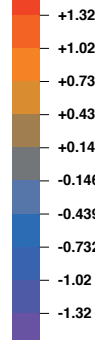

24 hours

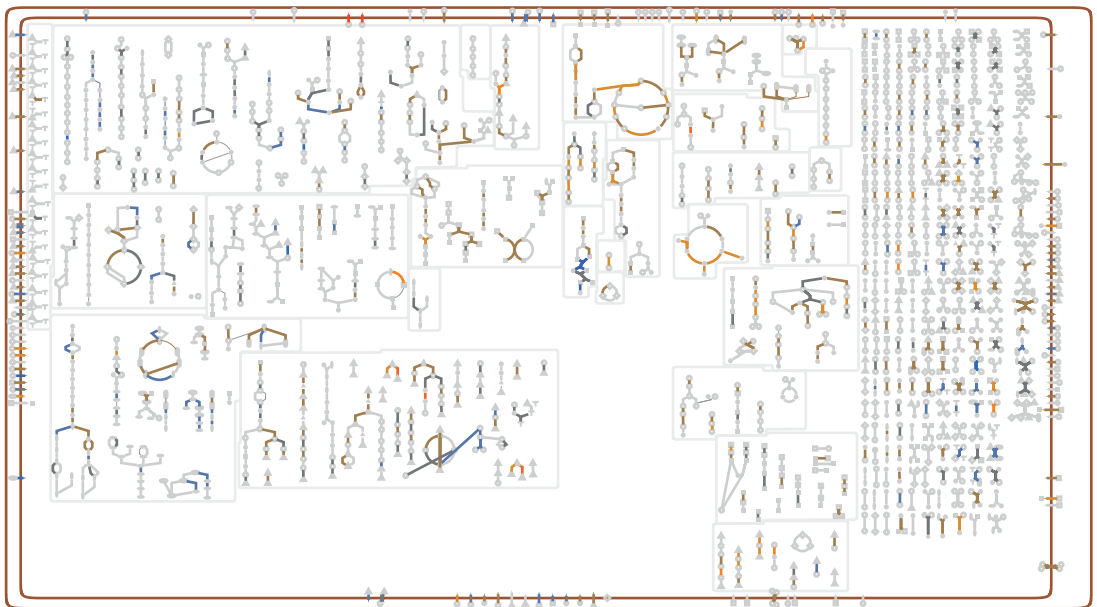

24h

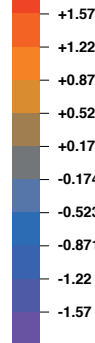

Essential genes

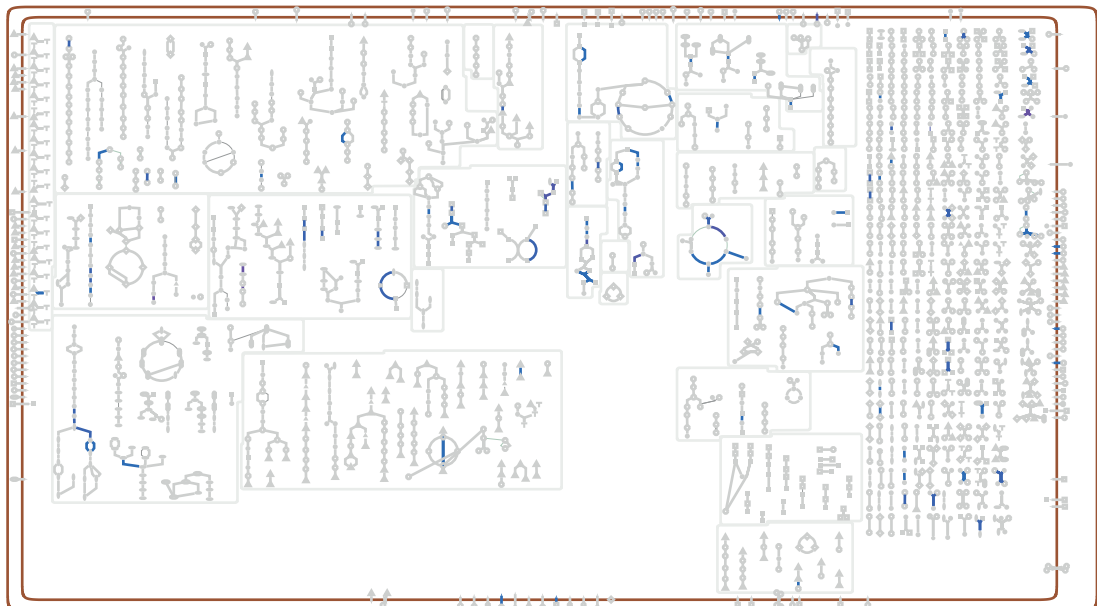

TraDIS

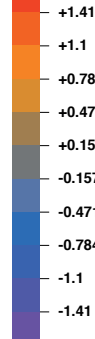

Supplement: Supplementary file 1 — Fig. S1. Metabolic pathways map showing changes in genes expression at 0.5, 6 and 24 h after entry into water. Genes encoding enzymes with significant change in expression ranging from ≤2‐fold (green) to ≥2‐fold (red) on entry into water. [file EMI-20-4079-s001.pdf]
